# Validation study on a non-linear dynamical model of the projectile

**DOI:** 10.1038/s41598-023-40619-2

**Published:** 2023-08-18

**Authors:** Jun Liang, Xuanhua Fan, Yuancen Wang, Shifu Xiao, Hongyong Chen

**Affiliations:** 1 Institute of Systems Engineering, China Academy of Engineering Physics, Mianyang, 621900 Sichuan China; 2 Mianyang Teachers’ College, Mianyang, 621000 Sichuan China

**Keywords:** Mechanical engineering, Nonlinear phenomena

## Abstract

Based on the theoretical approach of multi-body interaction dynamics, a theoretical model is constructed to simulate the nonlinear response amplification of the projectile structure. The accuracy and universality of the theoretical model were verified by comparing the response data calculated by the theoretical model with the experimental data. The results show that the theoretical model can predict the acceleration and strain response of the projectile structure more accurately, providing a non-linear dynamic analysis method for the projectile structure from the perspective of structural dynamics.

## Introduction

In order to improve the safety and environmental adaptability of the projectile during penetration, the typical projectile structure is usually assembled from multiple charge sections combined with partitions. During projectile impact and flight, multi-body interactions such as collisions and friction between the internal charge and the partitions are inevitable, leading to problems such as amplified structural dynamic response and premature local ignition^1–3^. The study of non-linear response patterns due to such multi-body interactions is important in assessing the structural safety of the projectile. In order to study the response patterns, an accurate and universal theoretical model of multi-body dynamics must first be constructed.

Research work at home and abroad in the field of projectile penetration is mainly focused on the number of penetration layers, depth prediction, study of the projectile overload curve, evaluation of the blast damage effect, target damage and protection, etc^4,5^. The relevant research is biased towards the field of impact dynamics. Studies of the intrusion penetration effect rarely consider the structural dynamic properties of the projectile structure itself. Amplification of the local dynamic response of the charge may be an important cause of charge ignition. In the study of the projectile structural dynamics, the correct description of the multi-body collision process and the construction of a reasonable dynamical model are important challenges for current research^6^. In the study on the dynamics of multi-body systems with clearances, Flores^7,8^ and others have done a lot of work, including systems with a single rotating sub-clearance and planar multi-body systems with multiple rotating sub-clearances, and proposed a method for modelling multi-body systems. Muvengei^9^ and others introduced Lugre friction into a computational model to study the dynamics of a planar multi-body system containing two rotating sub-clearances. Based on the generalised Hertz contact theory, Shi^10^ established a vibration model for a roll containing a symmetrical clearance, which was numerically solved using the 4th Runge–Kutta method to investigate the non-linear vibration characteristics between the roll and the guide plate. Chen^11^ introduced clearance, contact force and contact deformation into the dynamical equations to improve the modelling and numerical simulation accuracy, and studied the numerical integration methods. An^12^ has analysed three different types of models in order to clarify the basis for the selection of contact collision models for the dynamics simulation of multi-body system. Dong^13^ proposed a modelling method for the collision problem of multi-body systems with finite element discretization, discussed the treatment of the conditions of contact and separation, and finally verified the correctness of the method by comparing the numerical and theoretical solutions with one-dimensional collision problem as an example.

In this paper, we construct a multi-body interaction dynamics model based on the nonlinear vibration multi-body interaction theory, taking into account the interface non-linearity caused by clearances and collisions. On the basis of the theoretical derivation and simplification, the system of ordinary differential equations for the theoretical model is solved numerically by writing a 4th Runge–Kutta method calculation program. A multi-body interaction experiment is designed to match the theoretical model for the configuration structure under typical load excitation. By arranging acceleration sensors and high-sensitivity strain gauges, the acceleration and dynamic strain response signal data of the simulated projectile structure are obtained. The accuracy and universality of the theoretical model is verified by combining the theoretical and experimental calculation results.

## Calculation of the theoretical model

**Figure 1:**
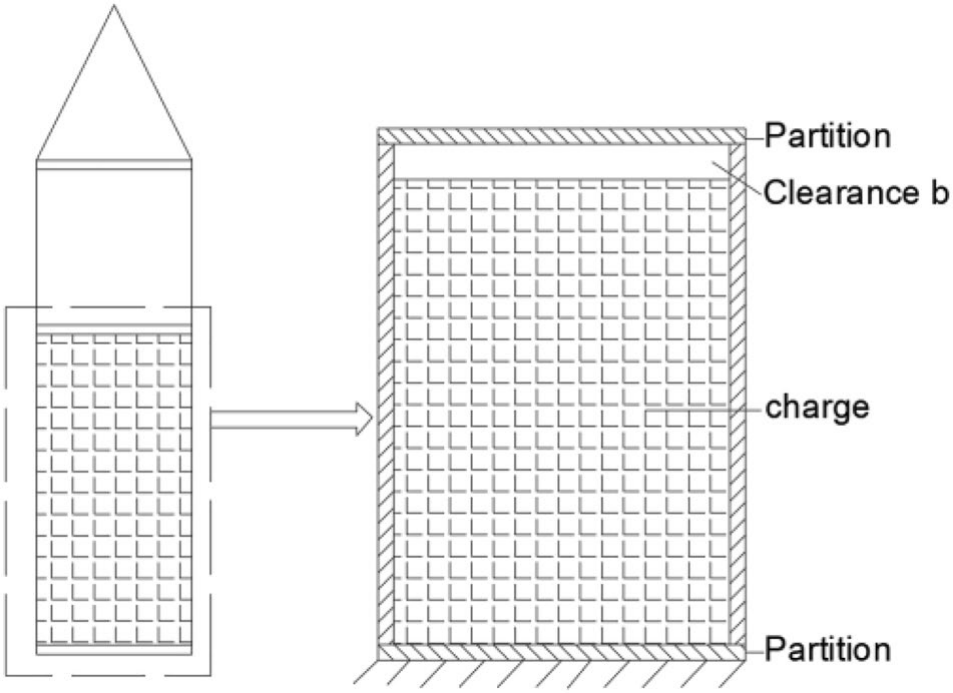
Illustrations of the simulated projectile.

**Figure 2:**
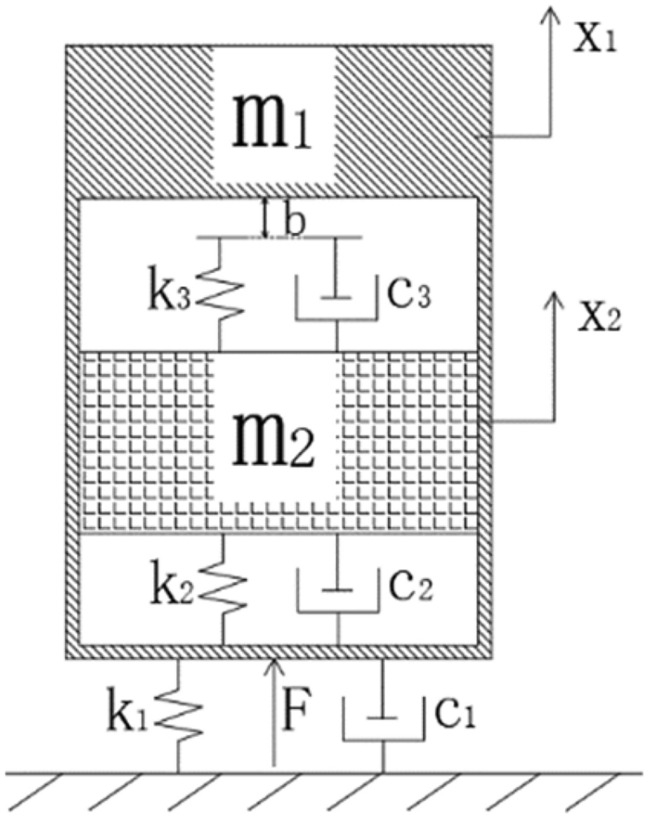
Theoretical model of the simulated projectile with clearance.

The dynamic response of the projectile during penetration consists of overall motion and local vibrations. Compared with the more rigid shell, the internal charge has a lower stiffness. Considering the flexible characteristics of the projectile and the interface non-linearity, a theoretical model of the local multi-body dynamics interaction between the shell and the charge is constructed, as shown in Fig. 1. The exterior is composed of a cylinder and partition simulating typical column characteristics. The interior is filled by a column simulating the structure of the charge. Mechanical vibration systems containing clearances and collisions are multi-parameter and high-dimensional systems. The non-linearity and singularity caused by factors such as collisions or impacts make the system have strong non-linear dynamic characteristics. It is very difficult to consider all physical processes in collisions comprehensively in the study, so the collision conditions and collision processes need to be reasonably simplified^14^. A schematic diagram of the simplified model using the concentrated mass method is shown in Fig. 2, where the bottom of the charge is connected to the shell partition in contact and a small clearance b exists between the top of the charge and the shell partition.

According to the different states of motion between the charge and the shell, the two-degree-of-freedom dynamical motion equation of the multi-body interaction of a typical projectile structure are established with the shell and the charge as the study object respectively. In order to better reflect the non-linear effects in the vibration process of the projectile structure, a squared non-linear term is introduced before the velocity term. The specific dynamical model is as follows:

1 $$ \left\{ {\begin{array}{*{20}c} {m_{1} \ddot{x}_{1} + c_{1} \left( {\varepsilon - x_{1}^{2} } \right)\dot{x}_{1} + k_{{1}} x_{{1}} - k_{2} \left( {x_{2} - x_{1} } \right) - c_{2} \left[ {\left( {\sigma - x_{2}^{2} } \right)\dot{x}_{2} - \left( {\varepsilon - x_{1}^{2} } \right)\dot{x}_{1} } \right] - {\text{f}}_{x} \left( {x_{1} , x_{2} , \dot{x}_{1} , \dot{x}_{2} , } \right) = F} \\ {m_{2} \ddot{x}_{2} + k_{2} \left( {x_{2} - x_{1} } \right) + c_{2} \left[ {\left( {\sigma - x_{2}^{2} } \right)\dot{x}_{2} - \left( {\varepsilon - x_{1}^{2} } \right)\dot{x}_{1} } \right] + {\text{f}}_{x} \left( {x_{1} , x_{2} , \dot{x}_{1} , \dot{x}_{2} , } \right) = 0 } \\ \end{array} } \right. $$

2 $$ F = - \left( {m_{1} + m_{2} } \right)a\cos \left( {\omega t} \right) $$

3 $$ {\text{f}}_{x} \left( {x_{1} , x_{2} , \dot{x}_{1} , \dot{x}_{2} , } \right) = \left\{ {\begin{array}{*{20}l} {k_{3} \left( {x_{2} - x_{1} - b} \right) + c_{3} \left[ {\left( {\sigma - x_{2}^{2} } \right)\dot{x}_{2} - \left( {\varepsilon - x_{1}^{2} } \right)\dot{x}_{1} } \right],{ }} \hfill & {x_{2} - x_{1} > b} \hfill \\ {0,} \hfill & {0 \le x_{2} - x_{1} \le b} \hfill \\ {k_{3} \left( {x_{2} - x_{1} } \right) + c_{3} \left[ {\left( {\sigma - x_{2}^{2} } \right)\dot{x}_{2} - \left( {\varepsilon - x_{1}^{2} } \right)\dot{x}_{1} } \right],{ }} \hfill & {x_{2} - x_{1} < 0} \hfill \\ \end{array} } \right. $$

where: *m*_*1*_ is the total mass of the cylinder shells, approximately 7.2 kg. *m*_*2*_ is the mass of the charge, approximately 8.7 kg. *k*_*1*_ and *c*_*1*_ are the stiffness and damping coefficients of the shells respectively. *k*_*2*_ and *c*_*2*_ are the stiffness and damping coefficients of the charge respectively. *k*_*3*_ and *c*_*3*_ are the collision stiffness and damping coefficients of charge with the upper and lower shells during vibration respectively. *x*_*1*_ is the displacement of the shells. *x*_*2*_ is the displacement of charge. *t* is the time. $f_{x} \left( {x_{1} ,x_{2} ,\dot{x}_{1} ,\dot{x}_{2} ,} \right)$ is the non-linear force at clearance *b*. *F* is the excitation load applied to the projectile. *a* is the acceleration load amplitude. $\omega$ is the load frequency. $\varepsilon$ and ${ }\sigma$ are the non-linear damping factor.

Because the entire theoretical model is designed mainly for axial sinusoidal excitation of the projectile, we also consider only the axial aspects of each physical parameter in our calculations. According to the definition of axial tensile and compressive stiffness, *k*_*1*_, *k*_*2*_ are calculated and *c*_*1*_, *c*_*2*_ are found. The contact process between the charge and the shell is described based on the Hertz theory. *k*_*3*_ and *c*_*3*_ are calculated according to Hertz theory^10^. Hertz theory is a mathematical elastodynamic approach to deriving formulas for contact problems^15^.

4 $$ k_{1} = \frac{{E_{1} A_{1} }}{{L_{1} }},\;\;k_{2} = \frac{{E_{2} A_{2} }}{{L_{2} }} $$

5 $$ k_{3} = 2dE^{*} $$

6 $$ \frac{1}{{E^{*} }} = \frac{{1 - p_{1}^{2} }}{{E_{1} }} + \frac{{1 - p_{2}^{2} }}{{E_{2} }} $$

7 $$ c_{3} = \frac{{3\left( {1 - \eta^{2} } \right)}}{{4v_{0} }}k_{3} \delta $$

where: *E*_*1*_, *A*_*1*_ and *L*_*1*_ are the elastic modulus, force area and total length of the shell respectively. *E*_*1*_ = 70 GPa, *A*_*1*_ = 0.0155 m^2^, *L*_*1*_ = 0.37 m. *E*_*2*_, *A*_*2*_ and *L*_*2*_ are the elastic modulus, force area and total length of the charge respectively. *E*_*2*_ = 10 GPa, *A*_*2*_ = 0.0095 m^2^, *L*_*2*_ = 0.1996 m. *d* is the charge diameter, approximately 110 mm. *p*_*1*_ and *p*_*2*_ are the Poisson's ratio of the shell and the charge respectively. *p*_*1*_ = 0.3, *p*_*2*_ = 0.3. $v_{0}$ is the initial relative velocity at impact, $v_{0}$ = $\dot{x}_{2} - \dot{x}_{1}$. $\eta$ is recovery factor, $\eta = \frac{{\dot{x}_{2}^{\prime } - \dot{x}_{1}^{\prime } }}{{\dot{x}_{2} - \dot{x}_{1} }}$. $\delta$ is contact deformation amount, $\delta = x_{2} - x_{1} - b$. $\dot{x}_{1}$ is the velocity of the shell before impact. $\dot{x}_{2}$ is the velocity of the charge before impact. $\dot{x}_{1}^{\prime }$ is the velocity of the shell after impact. $\dot{x}_{2}^{\prime }$ is the velocity of the charge after impact. The experiment placed velocity sensors at the top of the charge and at the bottom of the top partition to test the velocity. Testing the velocities at both positions gives $v_{0} = 0.46\;\;{\text{mm/s, }}\;\eta = 0.84$.

The calculation gives *k*_*1*_ = 2.9 × 10^9^ N/m, *k*_*2*_ = 4.8 × 10^8^ N/m, *k*_*3*_ = 2.1 × 10^9^ N/m, $c_{3} = 0.48k_{3} \delta$. The damping factor *c*_*1*_ of shell is 0.002 N/(m/s). The damping factor *c*_*2*_ of charge is 0.06 N/(m/s).

Substitute into Eq. (3):

8 $$ {\text{f}}_{x} \left( {x_{1} , x_{2} , \dot{x}_{1} , \dot{x}_{2} , } \right) = \left\{ {\begin{array}{*{20}l} {k_{3} \left( {x_{2} - x_{1} - b} \right) + 0.48k_{3} \left( {x_{2} - x_{1} - b} \right)\left[ {\left( {\sigma - x_{2}^{2} } \right)\dot{x}_{2} - \left( {\varepsilon - x_{1}^{2} } \right)\dot{x}_{1} } \right],{ }} \hfill & {x_{2} - x_{1} > b} \hfill \\ {0,} \hfill & {{ }0 \le x_{2} - x_{1} \le b} \hfill \\ {k_{3} \left( {x_{2} - x_{1} } \right) + 0.48k_{3} \left( {x_{2} - x_{1} } \right)\left[ {\left( {\sigma - x_{2}^{2} } \right)\dot{x}_{2} - \left( {\varepsilon - x_{1}^{2} } \right)\dot{x}_{1} } \right],{ }} \hfill & {x_{2} - x_{1} < 0} \hfill \\ \end{array} } \right. $$

Equation (8) is substituted into the set of Eqs. (1), while Eqs. (9), (10) and (11) are obtained by normalising^16^ the set of equations (1) using $\frac{{c_{1} }}{{m_{1} }} = 2\xi_{1} \omega_{1} ,{ }t = \frac{\tau }{{\omega_{1} }},{ }\overline{\omega } = \frac{{\upomega }}{{{\upomega }_{1} }}$. $\omega_{1}$ is the inherent frequencies of the shell. $\xi_{1}$ is the modal damping ratio of the shell. $\omega_{1} = \sqrt {\frac{{k_{1} }}{{m_{1} }}} = 2.45\; \times \;10^{4} \left( \frac{1}{s} \right),\;\;\xi_{1} = 8.42 \times 10^{ - 9}$. Let $f = \frac{{ - \left( {m_{1} + m_{2} } \right)a}}{{m_{1} \omega_{1}^{2} }},{ }\beta = \frac{{k_{2} }}{{k_{1} }},{ }\tau = \frac{{k_{3} }}{{k_{1} }},{ }\mu = \frac{{m_{1} }}{{m_{2} }},{ }\alpha = \frac{{c_{2} }}{{c_{1} }}$:

When $x_{2} - x_{1} > b$, charge in contact with the upper shell:

9 $$ \left\{ {\begin{array}{*{20}l} {\ddot{x}_{1} + 2\xi_{1} \left( {\varepsilon - x_{1}^{2} } \right)\dot{x}_{1} + x_{1} - \beta \left( {x_{2} - x_{1} } \right) - 2\xi_{1} \alpha \left[ {\left( {\sigma - x_{2}^{2} } \right)\dot{x}_{2} - \left( {\varepsilon - x_{1}^{2} } \right)\dot{x}_{1} } \right] - \tau \left( {x_{2} - x_{1} - b} \right)} \hfill \\ { - 0.48\tau \omega_{1} \left( {x_{2} - x_{1} - b} \right)\left[ {\left( {\sigma - x_{2}^{2} } \right)\dot{x}_{2} - \left( {\varepsilon - x_{1}^{2} } \right)\dot{x}_{1} } \right] = f\cos \left( {\overline{\omega }\tau } \right)} \hfill \\ {\ddot{x}_{2} + 2\xi_{1} \alpha \mu \left[ {\left( {\sigma - x_{2}^{2} } \right)\dot{x}_{2} - \left( {\varepsilon - x_{1}^{2} } \right)\dot{x}_{1} } \right] + \beta \mu \left( {x_{2} - x_{1} } \right) + \tau \mu \left( {x_{2} - x_{1} - b} \right)} \hfill \\ { + 0.48\tau \mu \omega_{1} \left( {x_{2} - x_{1} - b} \right)\left[ {\left( {\sigma - x_{2}^{2} } \right)\dot{x}_{2} - \left( {\varepsilon - x_{1}^{2} } \right)\dot{x}_{1} } \right] = 0} \hfill \\ \end{array} } \right. $$

When $0 \le x_{2} - x_{1} \le {\text{b}}$, no contact between the charge and either the upper or lower shell:

10 $$ \left\{ {\begin{array}{*{20}l} {\ddot{x}_{1} + 2\xi_{1} \left( {\varepsilon - x_{1}^{2} } \right)\dot{x}_{1} + x_{1} - \beta \left( {x_{2} - x_{1} } \right) - 2\xi_{1} \alpha \left[ {\left( {\sigma - x_{2}^{2} } \right)\dot{x}_{2} - \left( {\varepsilon - x_{1}^{2} } \right)\dot{x}_{1} } \right] = f\cos \left( {\overline{\omega }\tau } \right)} \hfill \\ {\ddot{x}_{2} + 2\xi_{1} \alpha \mu \left[ {\left( {\sigma - x_{2}^{2} } \right)\dot{x}_{2} - \left( {\varepsilon - x_{1}^{2} } \right)\dot{x}_{1} } \right] + \beta \mu \left( {x_{2} - x_{1} } \right) = 0} \hfill \\ \end{array} } \right. $$

When $x_{2} - x_{1} < 0$, charge in contact with the lower shell:

11 $$ \left\{ {\begin{array}{*{20}l} {\ddot{x}_{1} + 2\xi_{1} \left( {\varepsilon - x_{1}^{2} } \right)\dot{x}_{1} + x_{1} - \beta \left( {x_{2} - x_{1} } \right) - 2\xi_{1} \alpha \left[ {\left( {\sigma - x_{2}^{2} } \right)\dot{x}_{2} - \left( {\varepsilon - x_{1}^{2} } \right)\dot{x}_{1} } \right] - \tau \left( {x_{2} - x_{1} } \right)} \hfill \\ { - 0.48\tau \left( {x_{2} - x_{1} } \right)\omega_{1} \left[ {\left( {\sigma - x_{2}^{2} } \right)\dot{x}_{2} - \left( {\varepsilon - x_{1}^{2} } \right)\dot{x}_{1} } \right] = f\cos \left( {\overline{\omega }\tau } \right)} \hfill \\ {\ddot{x}_{2} + 2\xi_{1} \alpha \mu \left[ {\left( {\sigma - x_{2}^{2} } \right)\dot{x}_{2} - \left( {\varepsilon - x_{1}^{2} } \right)\dot{x}_{1} } \right] + \beta \mu \left( {x_{2} - x_{1} } \right) + \tau \mu \left( {x_{2} - x_{1} } \right)} \hfill \\ { + 0.48\tau \mu \left( {x_{2} - x_{1} } \right)\omega_{1} \left[ {\left( {\sigma - x_{2}^{2} } \right)\dot{x}_{2} - \left( {\varepsilon - x_{1}^{2} } \right)\dot{x}_{1} } \right] = 0} \hfill \\ \end{array} } \right. $$

The Eqs. (9), (10) and (11) were discretized and solved numerically using the 4th Runge–Kutta method. To facilitate programming, the 2nd order control equations were first processed by reducing the order^17^.

Let $$\left\{ {\begin{array}{*{20}c} {u_{1} = x_{1} } \\ {u_{2} = \dot{x}_{1} } \\ \end{array} } \right.,{ }\left\{ {\begin{array}{*{20}c} {u_{3} = x_{2} } \\ {u_{4} = \dot{x}_{2} } \\ \end{array} } \right.,{ }$$.

When $$u_{3} - u_{1} > b$$:

12 $$ \left\{ {\begin{array}{*{20}l} {\dot{u}_{1} = \dot{x}_{1} = u_{2} } \hfill \\ {\dot{u}_{2} = \ddot{x}_{1} = f\cos \left( {\overline{\omega }\tau } \right) - 2\xi_{1} \left( {\varepsilon - u_{1}^{2} } \right)u_{2} - u_{1} + \beta \left( {u_{3} - u_{1} } \right) + 2\xi_{1} \alpha \left[ {\left( {\sigma - u_{3}^{2} } \right)u_{4} - \left( {\varepsilon - u_{1}^{2} } \right)u_{2} } \right]} \hfill \\ { + \tau \left( {u_{3} - u_{1} - b} \right) + 0.48\tau \omega_{1} \left( {u_{3} - u_{1} - b} \right)\left[ {\left( {\sigma - u_{3}^{2} } \right)u_{4} - \left( {\varepsilon - u_{1}^{2} } \right)u_{2} } \right]} \hfill \\ {\dot{u}_{3} = \dot{x}_{2} = u_{4} } \hfill \\ {\dot{u}_{4} = \ddot{x}_{2} = - 2\xi_{1} \alpha \mu \left[ {\left( {\sigma - u_{3}^{2} } \right)u_{4} - \left( {\varepsilon - u_{1}^{2} } \right)u_{2} } \right] - \beta \mu \left( {u_{3} - u_{1} } \right) - \tau \mu \left( {u_{3} - u_{1} - b} \right)} \hfill \\ { - 0.48\tau \mu \omega_{1} \left( {u_{3} - u_{1} - b} \right)\left[ {\left( {\sigma - u_{3}^{2} } \right)u_{4} - \left( {\varepsilon - u_{1}^{2} } \right)u_{2} } \right]} \hfill \\ \end{array} } \right. $$

When $$0 \le u_{3} - u_{1} \le b$$:

13 $$ \left\{ {\begin{array}{*{20}l} {\dot{u}_{1} = \dot{x}_{1} = u_{2} } \hfill \\ {\dot{u}_{2} = \ddot{x}_{1} = f\cos \left( {\overline{\omega }\tau } \right) - 2\xi_{1} \left( {\varepsilon - u_{1}^{2} } \right)u_{2} - u_{1} + \beta \left( {u_{3} - u_{1} } \right) + 2\xi_{1} \alpha \left[ {\left( {\sigma - u_{3}^{2} } \right)u_{4} - \left( {\varepsilon - u_{1}^{2} } \right)u_{2} } \right]} \hfill \\ {\dot{u}_{3} = \dot{x}_{2} = u_{4} } \hfill \\ {\dot{u}_{4} = \ddot{x}_{2} = - 2\xi_{1} \alpha \mu \left[ {\left( {\sigma - u_{3}^{2} } \right)u_{4} - \left( {\varepsilon - u_{1}^{2} } \right)u_{2} } \right] - \beta \mu \left( {u_{3} - u_{1} } \right)} \hfill \\ \end{array} } \right. $$

When $$u_{3} - u_{1} < 0$$:

14 $$ \left\{ {\begin{array}{*{20}l} {\dot{u}_{1} = \dot{x}_{1} = u_{2} } \hfill \\ {\dot{u}_{2} = \ddot{x}_{1} = f\cos \left( {\overline{\omega }\tau } \right) - 2\xi_{1} \left( {\varepsilon - u_{1}^{2} } \right)u_{2} - u_{1} + \beta \left( {u_{3} - u_{1} } \right) + 2\xi_{1} \alpha \left[ {\left( {\sigma - u_{3}^{2} } \right)u_{4} - \left( {\varepsilon - u_{1}^{2} } \right)u_{2} } \right]} \hfill \\ { + \tau \left( {u_{3} - u_{1} } \right) + 0.48\tau \omega_{1} \left( {u_{3} - u_{1} } \right)\left[ {\left( {\sigma - u_{3}^{2} } \right)u_{4} - \left( {\varepsilon - u_{1}^{2} } \right)u_{2} } \right]} \hfill \\ {\dot{u}_{3} = \dot{x}_{2} = u_{4} } \hfill \\ {\dot{u}_{4} = \ddot{x}_{2} = - 2\xi_{1} \alpha \mu \left[ {\left( {\sigma - u_{3}^{2} } \right)u_{4} - \left( {\varepsilon - u_{1}^{2} } \right)u_{2} } \right] - \beta \mu \left( {u_{3} - u_{1} } \right) - \tau \mu \left( {u_{3} - u_{1} } \right)} \hfill \\ { - 0.48\tau \mu \omega_{1} \left( {u_{3} - u_{1} } \right)\left[ {\left( {\sigma - u_{3}^{2} } \right)u_{4} - \left( {\varepsilon - u_{1}^{2} } \right)u_{2} } \right]} \hfill \\ \end{array} } \right. $$

**Figure 3:**
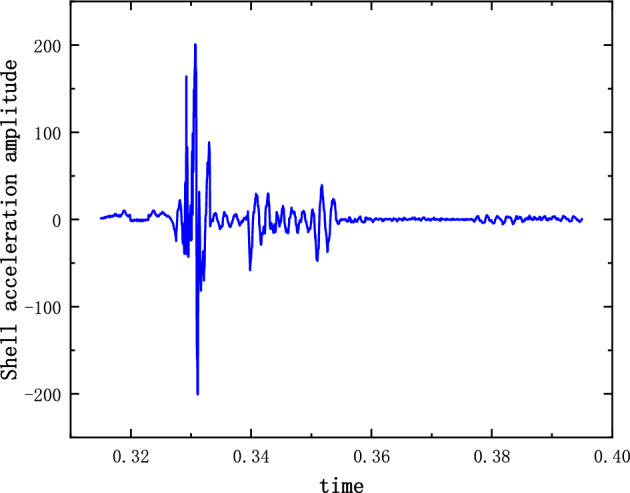
($\dot{u}_{2}$) Shell acceleration response.

**Figure 4:**
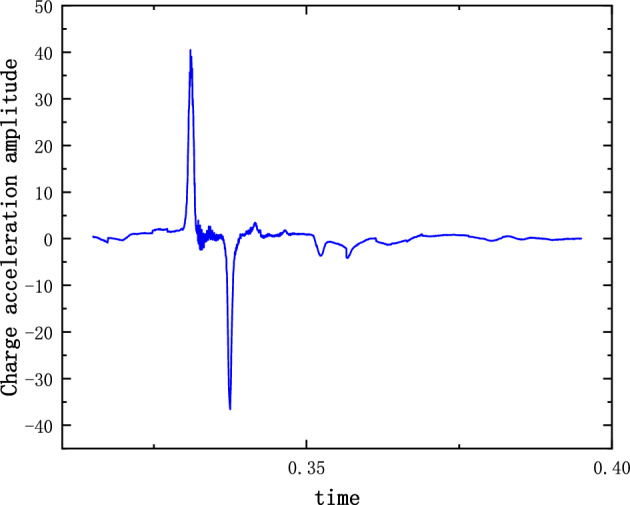
($\dot{u}_{4}$) Charge acceleration response.

**Figure 5:**
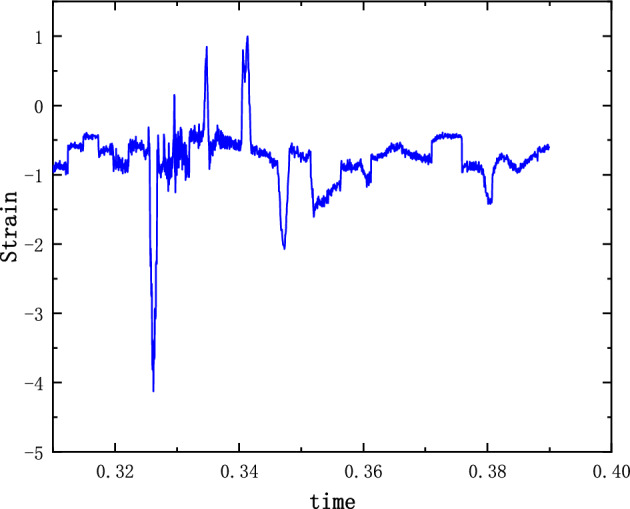
$(u_{3}$) Charge strain diagram.

The system of Eqs. (12), (13) and (14) was solved by the 4th Runge–Kutta method. The stiffness and damping parameters are substituted into the previous calculations. The non-linear damping coefficients are estimated based on the material parameters and the model structure. $\varepsilon$ and $\sigma$ are modified by combining the experimental model parameters. The modified values are 1.02 and 1.03 respectively. The displacement and acceleration amplitude response of the shell and charge are calculated. Figures 3 and 4 show the time domain diagrams of the acceleration response amplitude of the shell and charge respectively. Figure 5 shows the strain diagram of the charge. It can be seen from the figures that in the case of external load excitation, because there is a clearance in the structure, the charge collides with the upper and lower shells. The multi-body interaction between the charge and the shell is mutually transformed in the time domain, resulting in the amplification of the response amplitude. The amplification of the charge response amplitude is a potential factor leading to charge damage or ignition.

**Figure 6:**
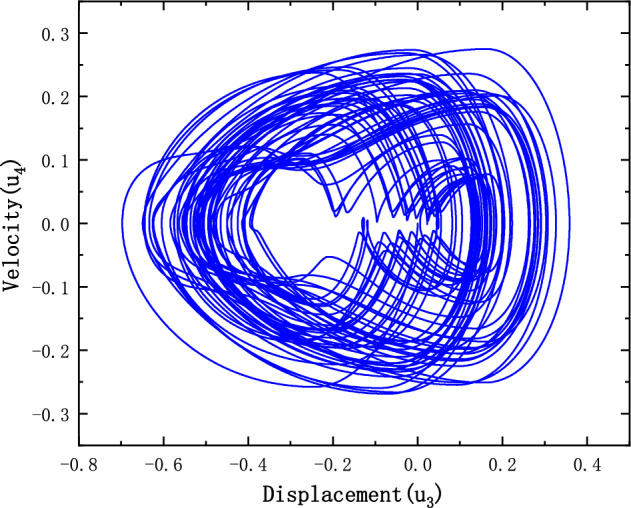
Phase space of the charge.

**Figure 7:**
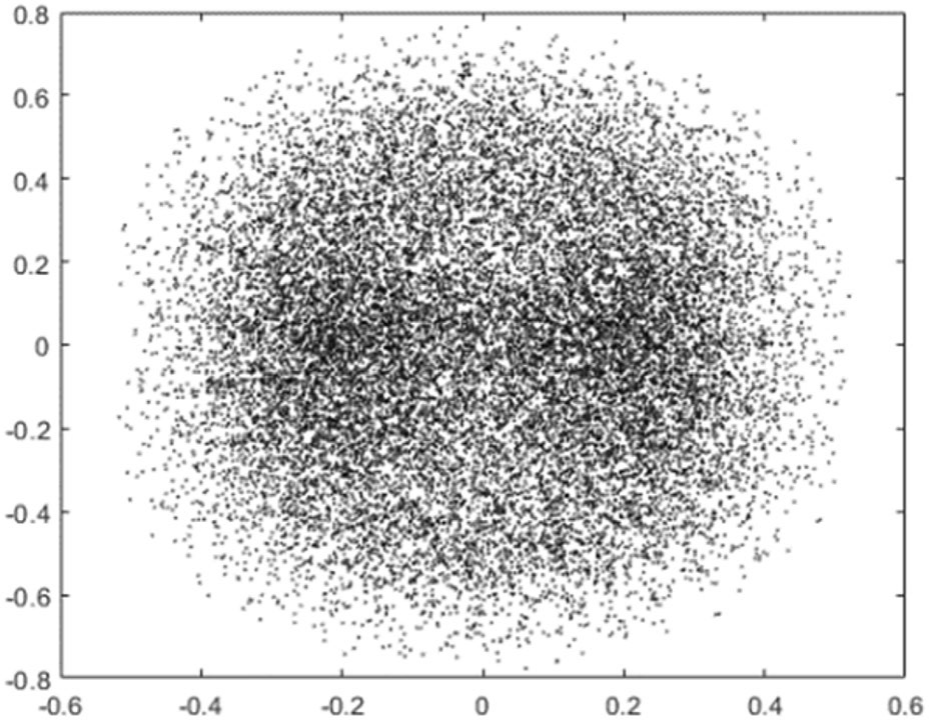
Poincare cross section of the projectile.

Figure 6 shows the phase plane of the charge. The trajectory lines are crossed. The structure of the charge has the typical characteristics of a high-dimensional and non-linear system where the motion is convergent. Figure 7 shows a poincare cross section of the simulated projectile, where the shape of the motion can be succinctly determined from the intersection of the rail lines with the cross section. The motion of the projectile containing the clearance in phase space exhibits quasi-periodic rotation after rotation most of the time, but there are also patches of dense points and a hierarchical structure at the edge sections, indicating that chaotic conditions also exist in the motion.

## Comparison of experimental and theoretical calculation results

5 g half-sine vibration tests were carried out for a cylindrical structure, as shown in Table 1. Combined with the theoretical model of multi-body interaction, a data acquisition and analysis system is applied to measure the acceleration time-domain signal at each measurement point. Acceleration response amplification data of key components are obtained. The corresponding responses were analysed. A sinusoidal excitation load is applied to the bottom of the projectile. The load inherent frequency is 1500 Hz. The load amplitude is 5 g.

**Table 1:** Half-sine vibration test conditions.

Test subject	Load inherent frequency/Hz	Amplitude/g	Testing time/ms
Two-layer barrel	1500	5	0–80

**Figure 8:**
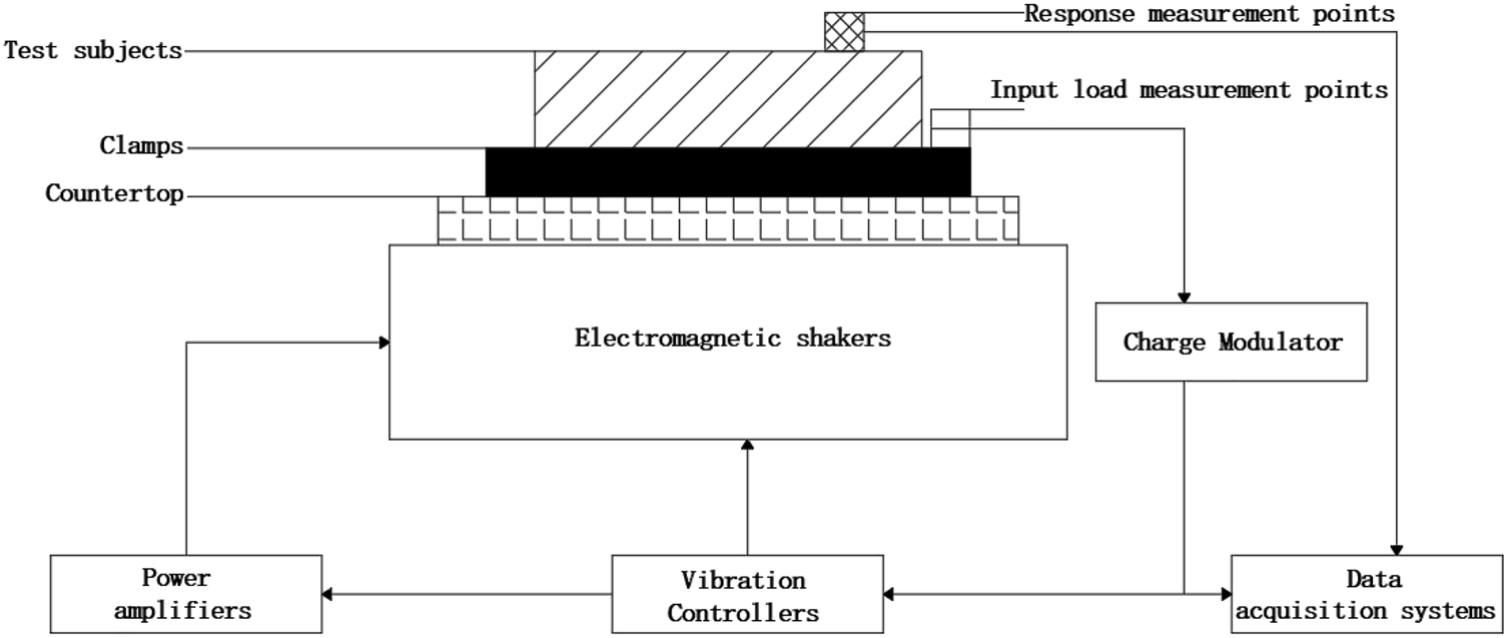
Vibration test system configuration.

**Figure 9:**
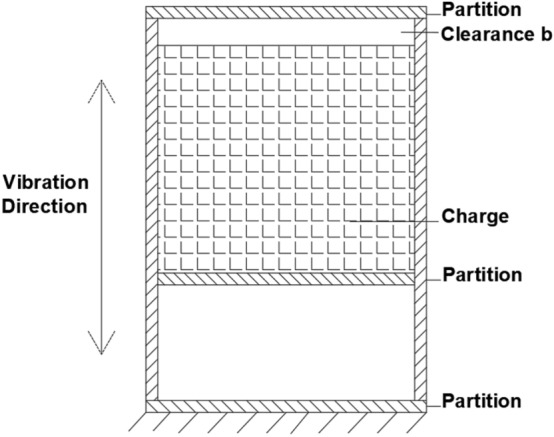
Simplified diagram of test objects.

The configuration of the test and test system is shown in Fig. 8, simplifying the component measurement items by collecting the input load and the acceleration response time domain signals. A simplified diagram of the test subject is shown in Fig. 9.

**Figure 10:**
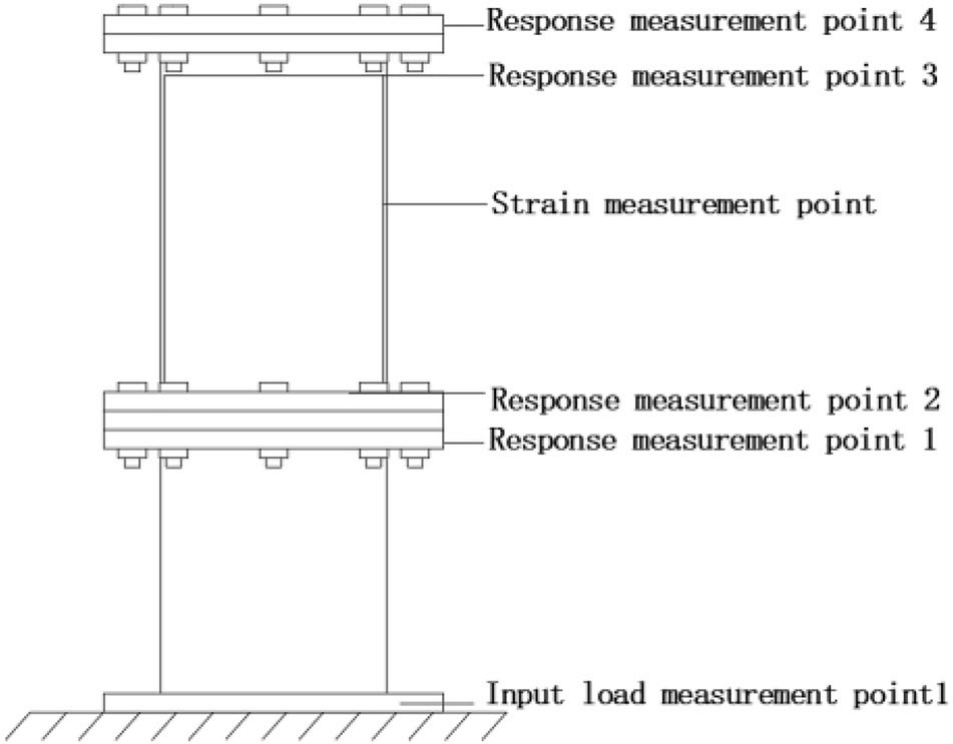
Test point layout.

Acceleration sensors were arranged at the bottom of the shell, the bottom of the charge, the top of the charge and the top of the shell respectively. Strain gauges were arranged at the centre of the side of the charge. The measurement directions are all in the axial direction of the simulated projectile, as shown in Fig. 10. The acceleration and strain responses of the simulated charge structure were obtained by applying axial half-sine impact load excitation to the bottom end of the experimental part structure, and combined with the calculation results of the theoretical model to verify the accuracy and universality of the theoretical model.

**Figure 11:**
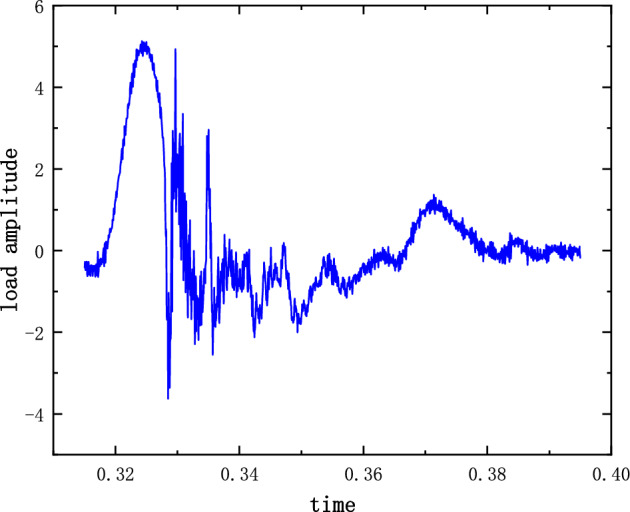
Experimental excitation load.

Figure 11 shows a graph of the excitation load for the experiment. The half-sine vibration experiment is used as a reference for comparing the theoretical model simulation results with the experimental test results. The key parameters of the multi-body interaction theoretical model are revised mainly based on the 5 g half-sine impact experimental results.

**Figure 12:**
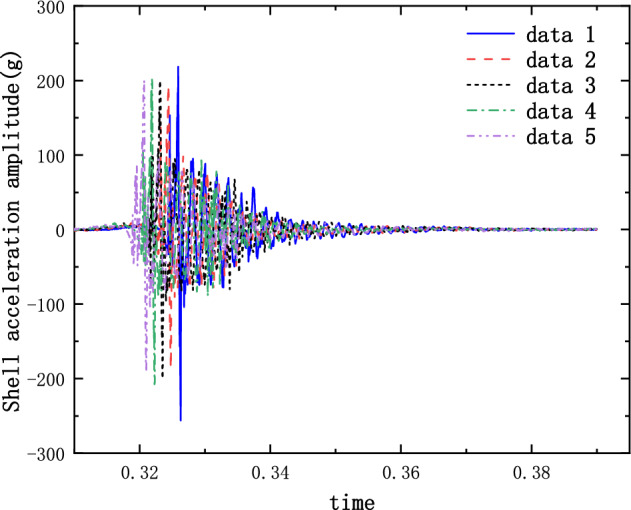
Shell acceleration response.

**Figure 13:**
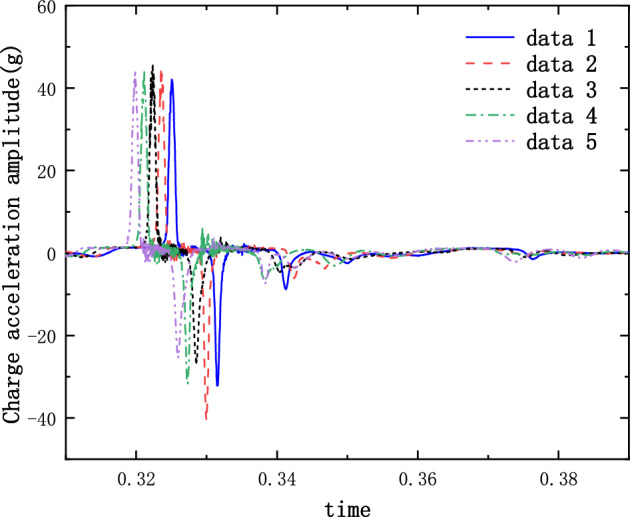
Charge acceleration response.

**Figure 14:**
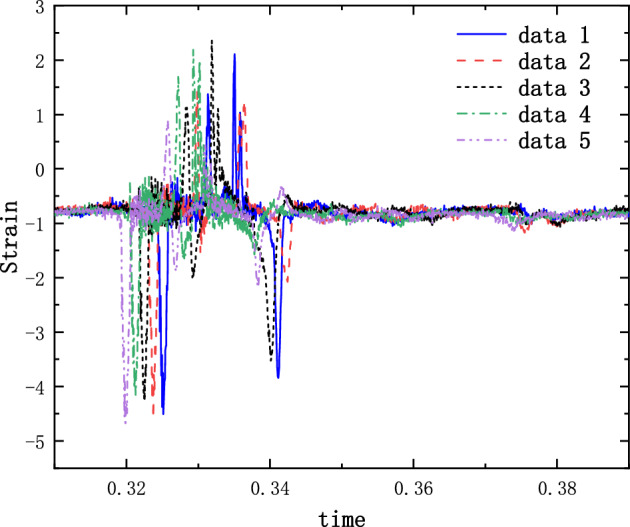
Charge strain diagram.

**Figure 16:**
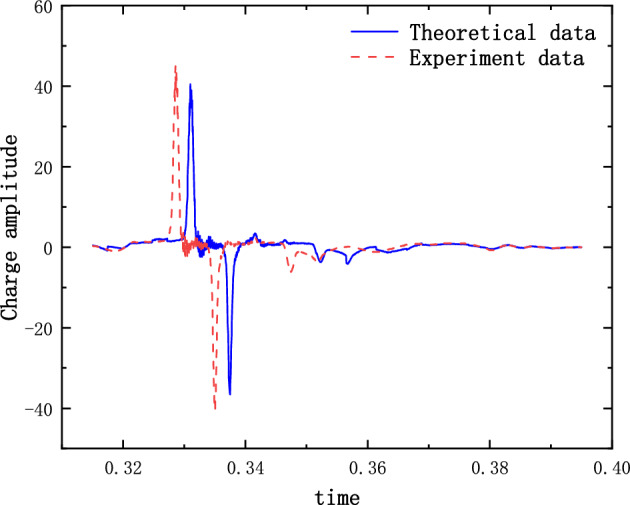
Comparison of charge acceleration.

**Figure 17:**
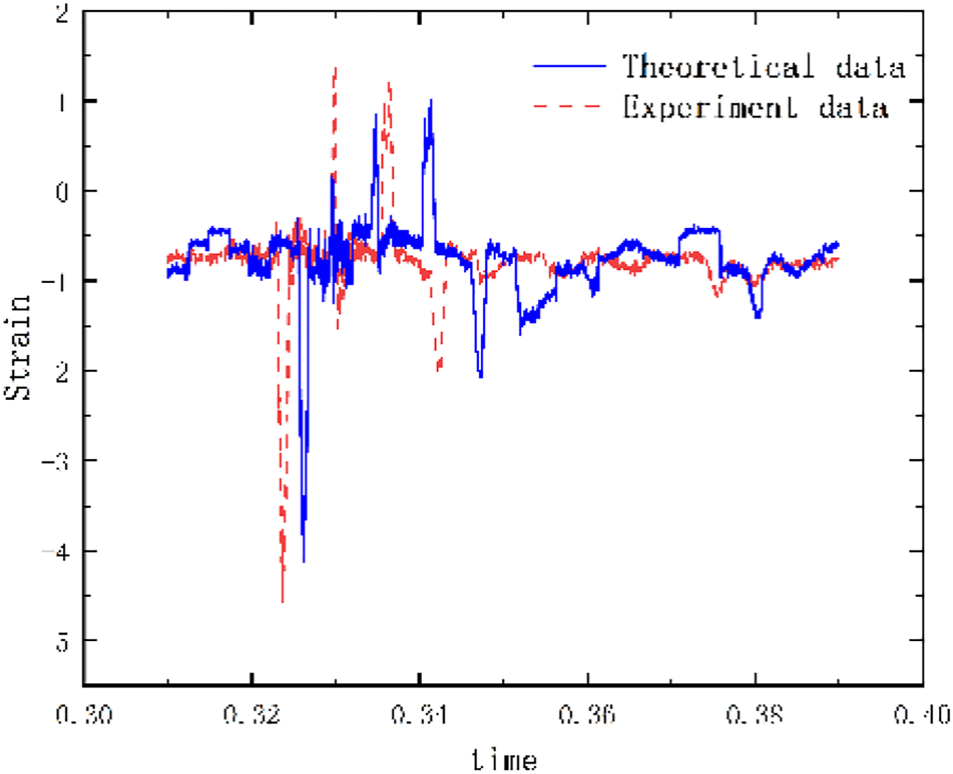
Comparison of charge strain.

The results of the 5 g half-sine impact experiments are shown in Figs. 12, 13 and 14. Figure 12 shows the acceleration measurement signal of the shell in multiple experiments. Figures 13 and 14 show the acceleration and strain measurement signals of the charge in multiple experiments. At the start of the half-sine impact, the charge results in a collision with the top partition of the shell and then returns to collide with the bottom partition, which in turn shocks and decays. Because of the linear condition, the parameter of the nonlinear term takes the value of 1. Considering the effect of nonlinear perturbation, the parameter takes the value around 1. Our preliminary values range from − 0.95 to 1.05. Combined with our multiple sets of experimental data, we finally selected 1.02 and 1.03 as the parameter values in the theoretical model. With this parameter value, the theoretical model can predict the structural vibration response under different experimental conditions more accurately. After correction of the non-linear damping coefficients, the acceleration and strain responses of the simulated charge structure under 5 g half-sine impact conditions were calculated and compared with the experimental test results are shown in Figs. 15, 16 and 17. The second set of experimental data selected from the five sets of data in Figs. 12, 13 and 14 is compared with the theoretical calculations.

**FFigure 15:**
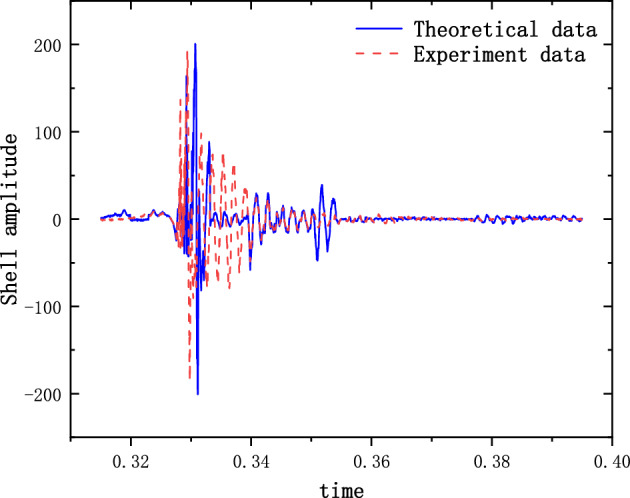
Comparison of shell acceleration.

From Figs. 15, 16 and 17, it can be seen that the acceleration results of theoretical calculations and experimental tests follow the same main regular trend. There are some differences in the comparison results because the stiffness and damping coefficients are difficult to obtain accurately and some of the non-linear factors cannot be fully taken into account. Similarly, the graphs do not overlap well in time as seen in the comparative figures of the theoretical and experimental results, as there is some error in the time tested during the experiment. The deviation from the maximum value of acceleration is less than 10% when comparing the theoretical calculation with the maximum mean value of experimental test results. The deviation from the maximum value of strain is also less than 10%. The results demonstrate that the constructed two-degree-of-freedom theoretical model can predict the acceleration and strain response of the simulated projectile structure more accurately.

## Conclusion

This paper constructs a theoretical dynamical model for the amplification of the non-linear response of a simulated projectile structure based on the theory of non-linear vibrational multi-body interactions, taking into account the interface non-linearities. Model modification and validation are completed by designing and conducting relevant multi-body interaction experiments to achieve accurate prediction of response amplification of the projectile structure by the non-linear dynamical theoretical model. The calculated results of the two-degree-of-freedom and non-linear dynamical theoretical model constructed in this paper are in general agreement with the trends of the main response variation patterns of the 5 g half-sine vibration experimental test results. The maximum peak and mean deviations for both theoretical and experimental comparisons of acceleration and strain are less than 10%. The dynamical theory model constructed in this paper has wide applicability. This will be followed by an analysis of several system parameters that have a significant influence on the dynamical behaviour of the system, to develop a fine predictive capability of the dynamical response of complex projectile structures and to understand the possible causes of premature ignition or explosion of the projectile during penetration.

## Data Availability

The datasets generated and analysed during the current study are not publicly available due the data also forms part of an ongoing study but are available from the corresponding author on reasonable request.
